# Supplementary material for: Empirical methods for controlling false positives and estimating confidence in ChIP-Seq peaks
Source: BMC Bioinformatics. 2008 Dec 5;9:523. doi: 10.1186/1471-2105-9-523 (PMC2628906; doi:10.1186/1471-2105-9-523)
Supplement: Additional File 1 — A variety of html documents from the USeq web site detailing the available applications, their best usage, output file type descriptions, command line menus, etc. [file 1471-2105-9-523-S1.zip › USeqUserGuides/JavaDoc.zip!/JavaDoc/trans/main/Oligo.html]

Oligo


|  |  |  |
| --- | --- | --- |
| **Overview** \| **Package** \| **Class** \| **Use** \| **Tree** \| **Deprecated** \| **Index** \| **Help** | |  |
| **PREV CLASS**   **NEXT CLASS** | **FRAMES**    **NO FRAMES**     **All Classes** |  |
| SUMMARY: NESTED \| FIELD \| CONSTR \| METHOD | DETAIL: FIELD \| CONSTR \| METHOD |  |


---


## trans.main Class Oligo

```
java.lang.Object
  trans.main.Oligo
```

**All Implemented Interfaces:**: java.io.Serializable

---

``` public class Oligo extends java.lang.Object implements java.io.Serializable ```

Container to hold information about a particular oligo.

**See Also:**: Serialized Form

---

**Constructor Summary**

`Oligo(int index,
int start,
int hits,
java.util.ArrayList intensities,
java.lang.String sequence)`


| **Method Summary** | |
| --- | --- |
| `float[]` | `getControlIntensities(int numControlInt)` |
| `int` | `getIndex()` |
| `java.util.ArrayList` | `getIntensities()` |
| `double` | `getLeftSlope()` |
| `int` | `getMatches()` |
| `double` | `getRightSlope()` |
| `java.lang.String` | `getSequence()` |
| `double` | `getSmoothedScore()` |
| `int` | `getStart()` |
| `float[]` | `getTreatmentIntensities(int numTreatInt)` |
| `boolean` | `isInPeak()` |
| `void` | `setInPeak(boolean inPeak)` |
| `void` | `setLeftSlope(double leftSlope)` |
| `void` | `setRightSlope(double rightSlope)` |
| `void` | `setSequence(java.lang.String sequence)` |
| `void` | `setSkip(boolean skip)` |
| `void` | `setSmoothedScore(double smoothedScore)` |
| `boolean` | `skip()` |
| `java.lang.String` | `toString()` |
| `java.lang.String` | `toString(int numTreatInt, int numControlInt, java.lang.String divider)` |

**Methods inherited from class java.lang.Object**

`equals, getClass, hashCode, notify, notifyAll, wait, wait, wait`

**Constructor Detail**

### Oligo

```
public Oligo(int index,
             int start,
             int hits,
             java.util.ArrayList intensities,
             java.lang.String sequence)
```


**Method Detail**

### toString

```
public java.lang.String toString()
```

:   **Overrides:**: `toString` in class `java.lang.Object`

---


### toString

```
public java.lang.String toString(int numTreatInt,
                                 int numControlInt,
                                 java.lang.String divider)
```

---


### getTreatmentIntensities

```
public float[] getTreatmentIntensities(int numTreatInt)
```

---


### getControlIntensities

```
public float[] getControlIntensities(int numControlInt)
```

---


### getStart

```
public int getStart()
```

---


### getIntensities

```
public java.util.ArrayList getIntensities()
```

---


### getIndex

```
public int getIndex()
```

---


### getSequence

```
public java.lang.String getSequence()
```

---


### getSmoothedScore

```
public double getSmoothedScore()
```

---


### setSmoothedScore

```
public void setSmoothedScore(double smoothedScore)
```

---


### isInPeak

```
public boolean isInPeak()
```

---


### setInPeak

```
public void setInPeak(boolean inPeak)
```

---


### skip

```
public boolean skip()
```

---


### setSkip

```
public void setSkip(boolean skip)
```

---


### getLeftSlope

```
public double getLeftSlope()
```

---


### setLeftSlope

```
public void setLeftSlope(double leftSlope)
```

---


### getRightSlope

```
public double getRightSlope()
```

---


### setRightSlope

```
public void setRightSlope(double rightSlope)
```

---


### getMatches

```
public int getMatches()
```

---


### setSequence

```
public void setSequence(java.lang.String sequence)
```


---


|  |  |  |
| --- | --- | --- |
| **Overview** \| **Package** \| **Class** \| **Use** \| **Tree** \| **Deprecated** \| **Index** \| **Help** | |  |
| **PREV CLASS**   **NEXT CLASS** | **FRAMES**    **NO FRAMES**     **All Classes** |  |
| SUMMARY: NESTED \| FIELD \| CONSTR \| METHOD | DETAIL: FIELD \| CONSTR \| METHOD |  |


---
